# Exploration of the mechanism of aloin ameliorates of combined allergic rhinitis and asthma syndrome based on network pharmacology and experimental validation

**DOI:** 10.3389/fphar.2023.1218030

**Published:** 2023-09-14

**Authors:** Yan Feng, Han Qiao, Hongyun Liu, Jvfei Wang, Huaping Tang

**Affiliations:** ^1^ Department of Respiratory Medicine, Qingdao University, Qingdao, China; ^2^ Department of Pathology, Qingdao Hospital, University of Health and Rehabilitation Sciences (Qingdao Municipal Hospital), Qingdao, China; ^3^ Department of Respiratory Medicine, Qingdao Hospital, University of Health and Rehabilitation Sciences (Qingdao Municipal Hospital), Qingdao, China

**Keywords:** aloin, combined allergic rhinitis and asthma syndrome, network pharmacology, molecular dynamics, MAPK signaling pathway

## Abstract

**Background:** Aloin, as a bioactive compound, has a variety of pharmacological functions, but its effects on combined allergic rhinitis and asthma syndrome (CARAS) have not been studied. To clarify the protective effect and mechanism of aloin in the treatment of CARAS by network pharmacology, molecular dynamics simulation and experiment.

**Methods:** The targets of aloin, allergic rhinitis and asthma were obtained from various databases. The protein interaction network was constructed for the common targets, and molecular docking and molecular dynamics simulations were performed for the core targets. Functional and pathway enrichment analysis of common targets was also performed using R software. Varieties of biological experiments were conducted to verify the effect of aloin on the inflammatory changes of CARAS and its regulatory mechanism.

**Results:** A total of 42 anti-allergic rhinitis and 58 anti-asthma targets were obtained, and 5 core anti-allergic rhinitis and 6 core anti-asthma targets were identified using topological analysis. GO and KEGG analyses showed that endopeptidase activity and MAPK signaling pathway played important roles in allergic rhinitis and asthma. Molecular docking and molecular dynamics simulations showed that aloin could stably bind to the core target proteins. Experimental verification showed that aloin significantly inhibited the expression of inflammatory factors, and may regulate CARAS by down-regulating MAPK signaling related proteins.

**Conclusion:** This study identified the protective effect, potential target and mechanism of aloin on CARAS. It provides reference for understanding the molecular mechanism and clinical application of aloin in the ameliorates of CARAS.

## Introduction

Asthma and allergic rhinitis (AR) frequently coexist, influencing each other’s disease course and severity (Leynaert et al., 2000; Brozek et al., 2017). The combined allergic rhinitis and asthma syndrome (CARAS) refers to the simultaneous clinical or subclinical hypersensitivity of the upper respiratory tract (AR) and the allergic symptoms of the lower respiratory tract (asthma) following the “one airway, one disease” principle (Grossman, 1997; Paiva Ferreira et al., 2019). It is characterized by an eosinophil-infiltrated type 2 immune response and the production of cytokines. For instance, Interleukin-5 (IL-5) is essential for airway eosinophilia, and Interleukin-4 (IL-4) and Interleukin-13 (IL-13) are necessary for Immunoglobulin E (IgE) production. IL-13 promotes hyperresponsiveness, mucus production, and airway fibrosis through tissue remodeling (Fahy, 2015; Wynn, 2015). These cytokines are responsible for producing allergenic specific IgE, eosinophilocytosis, and mast cell activation, releasing histamine, prostaglandins, and leukotrienes, promoting bronchoconstriction, and increasing vascular permeability, edema, vasodilatation, and airway hyperresponsiveness (Lambrecht et al., 2019; Hammad and Lambrecht, 2021). In addition, the pathogenesis of CARAS is directly related to atopic individuals, who are genetically susceptible to external allergens, which can be present in the air and can enter the respiratory tract, promoting the imbalance between innate and adaptive immune responses, thus causing the pathogenesis of CARAS (Eifan and Durham, 2016). The resulting clinical symptoms severely impact patients’ quality of life and impose a significant economic and social burden. Currently, non-specific therapies like allergen avoidance and immune regulation are commonly utilized, while antihistamines, glucocorticoids, mast cell stabilizers, leukotriene receptor antagonists and other drugs are commonly used for treatment. However, the clinical treatment effect is still suboptimal, relapse is expected, the long-term efficacy is poor, and it has severe side effects (Meltzer et al., 2013; Bel et al., 2014). Consequently, it is crucial to discover effective treatment methods.

Numerous herbal extracts are effective anti-inflammatory medications. Aloe vera is a medicinal plant with numerous biological effects, including liver protection (Chandan et al., 2007), blood sugar regulation (Pradeep et al., 2016), and wound healing promotion (Liang et al., 2021). Aloin is one of the primary bioactive substances extracted from aloe vera. Numerous studies have confirmed that aloe vera possesses numerous pharmacological properties. Anti-inflammatory (Luo et al., 2018), anti-tumor progression (Zhang et al., 2017), anti-osteoporosis (Pengjam et al., 2016), anti-pathogen (Lewis et al., 2022), metabolic regulation (Wang et al., 2020), and organ protection (Sun et al., 2021) are some of these effects. However, the role of aloin in the inflammatory response of CARAS has yet to be studied.

Based on network pharmacology, molecular docking, molecular dynamics simulation, and animal experiments, this study aims to investigate the effect of aloin on the inflammatory response of CARAS.

## Methods

### Target gene analysis of aloin

**FIGURE 1:**
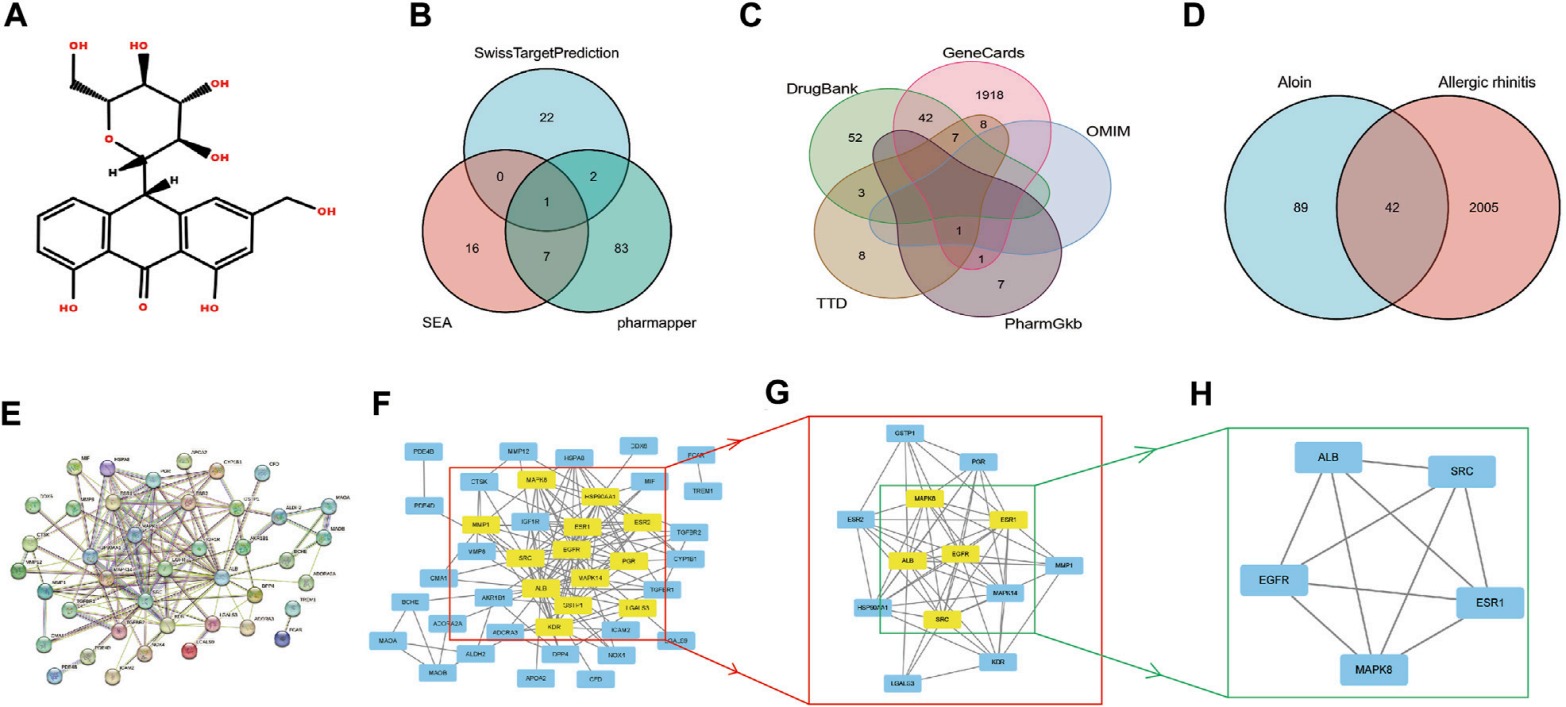
The network pharmacology analysis of aloin in the treatment of AR. **(A)** Chemical structural formula of aloin. **(B)** Potential predicted targets of aloin. **(C)** Potential predictive targets for AR. **(D)** Aloin and AR common targets. **(E)** PPI network of common targets. **(F–H)** Topological analysis of the common targets.

Figure 1A displays the aloin molecular structure obtained from the PubChem database. The target genes of aloin were subsequently identified through a search of the Swiss Target Prediction database, the Pharm Mapper database, and the Similarity Ensemble Approach database. Eliminating duplication, the potential targets were identified by combining the target genes obtained from the three databases.

### Target analysis of AR and asthma

We used “allergic rhinitis” and “asthma” as keywords to search and screen disease-related genes in the Gene Cards, Online Mendelian Inheritance in Man (OMIM), Therapeutic Target Database (TTD), Drug Bank, and Pharm Gkb databases. As a result, we obtained the disease targets for them, respectively. The intersections were then imported into a Venn diagram to determine the intersection targets for the treatment of AR and asthma.

### Construction of interaction network of aloin anti-AR and asthma target proteins

The intersection target genes were imported into the String database, organism was limited to “*Homo sapiens*,” medium confidence was set to >0.4, the node was hidden where the network broke open the connection, and the interrelationship diagram of the target proteins was obtained. Then, Cytoscape 3.9.0 software was imported to perform basic network topology analysis using Cyto NCA plug-in. The Eigenvector selected as betweenness (BC), closeness (CC), degree (DC), eigenvector (EC), network (NC), and Local Average Connections-based (LAC) targets above the mean value of method were taken as the key core targets; core components were obtained by analysis.

### Molecular docking verification of aloin and core targets

The universal Protein Resource (Uniprot) and Protein Data Bank (PDB) databases were searched for the three-dimensional structure of the key core target of aloin in asthma and allergic rhinitis, respectively. First, we downloaded the crystal structures of six central targets from the PDB library, including SRC (1o43), ALB (1n5u), EGFR (1xkk), ESR1 (1sj0), MAPK8 (2xrw), and KDR (1ywn). Then, using Chem3D software, the 2D structure of a molecular ligand was converted to its 3D structure. Next, Auto Dock Tools and Vina were used to dehydrate, hydrogenate, and charge core target proteins before performing molecular docking with aloin. Finally, mapping was performed with the PyMOL software.

### Molecular dynamics simulation

CHARMM36M was used to describe the force field of proteins. The system’s solvent was represented by the TIP3P model, Na+ and Cl-were used to balance the system’s charge, and the complex’s topology file was generated with Gromacs’ pdb2gmx module. The generalized Amber force field (GAFF) and AM1-BCC determined the ligand’s parameters and charge. First, the energy of the solvated system was minimized, and then this process was repeated 5,000 times to optimize the entire system. The entire system was then heated using the canonical ensemble (NVT) system. The system was slowly heated from 0 to 310 K in 100 ps by applying a harmonic constraint of 2 kcal/mol/A2 force constant to the protein skeleton. After heating, the time step was set to 2 fs, and 100 ps was first balanced in the NVT ensemble. Temperature and pressure were controlled using the Langevin and Berendsen algorithms, so the temperature was maintained at 310 K, and the pressure was maintained at 1 atm. Using Gromacs-2022.04GPU for molecular dynamics simulation, The root mean square deviation (RMSD), Root mean square fluctuation (RMSF), The radius of gyration (Rg), Hydrogen bond (Hbond), and solvent accessible surface (SASA) of the composite were calculated. There were 50,000,000 nsteps, and 100 ns were simulated.

### Gene ontology (GO) and Kyoto encylopaedia of genes and genomes path (KEGG) enrichment analysis

R software 4.2.1 was used to perform GO enrichment analysis of intersection targets, including the analysis of three modules: Biological process (BP), Cellular component (CC), and Molecular function (MF). Using *p* < 0.05 as the criterion, the top ten targets were chosen based on the number of targets involved. Simultaneously, KEGG pathway analysis for intersection targets was conducted. The screening criterion was *p* < 0.05, and the top 30 signals were selected based on this criterion in order to eradicate signal pathways with a high enrichment degree.

### Experimental animals

Female BALB/c mice aged 6–8 weeks, Specific pathogen Free (SPF) grade, and weighing 20 ± 2.5 g were bred in the Animal Room. Throughout the experiment, mice were maintained at a temperature between 23°C and 25°C with a 12-h light-dark cycle and unrestricted access to water and food. The mice used in this study were bred according to the protocol approved by the animal welfare ethics committee of Qingdao University (No.: 220203BALB/C54202208073).

### Grouping and handling of animals

The experimental animals were randomly divided into six groups (n = 6 each): normal group, CARAS group (OVA sensitization and challenge), aloin (≥98% purity, Shanghai Yuanye Biotechnology Co., Ltd.) low-dose group (10 mg/kg), aloin medium-dose group (20 mg/kg), aloin high-dose group (40 mg/kg), and budesonide (BUD, Shanghai Astrazeneca Pharmaceutical Co., Ltd.) group (0.9 mg/kg, BUD group). On days 0 and 7, All mices were sensitized intraperitoneally with 50 μg/mL ovalbumin (OVA, Sigma Company, US) and 10 mg/mL Aluminium hydroxide (Al(OH)_3_, Shanghai McLean Biochemical Technology Co., Ltd.) suspension of 0.2 mL. On three consecutive days a week for 3 weeks from day 21 to day 37, the treatment groups were intranasal instilled with aloin and budesonide at a dose of 20 μL in the bilateral nasal cavities of the mice by intranasal instillation, followed by 20 μL of OVA in the experimental group. On days 38–42, treatment groups were treated with aloin or budesonide 1 h before OVA challenge, and then the experimental group were aerosolized with OVA (5%) for 30 min, while the mice in the control group received PBS. On day 43, mice were euthanized by cervical dislocation, and biological materials such as lung tissue, nasal mucosa tissue, serum, and alveolar lavage fluid were collected for subsequent analysis (Li et al., 2016; Tang et al., 2019).

### Inflammatory symptoms and histopathology

After the last OVA challenge (day 42), each animal was observed for 10 min for symptoms such as nose-picking and sneezing. Before embedding the paraformaldehyde-fixed tissue in paraffin, the tissue was dehydrated with ethanol. The nasal and lung sections were then cut (three to five μm) using a microtome and stained with hematoxylin-eosin staining (H&E). The thickness of nasal mucosa and the number of eosinophils were measured quantitatively in a blinded manner, and the severity of peribronchial inflammation was scored as 0, normal; 1, few cells; 2, inflammatory cell ring 1 cell layer deep; 3, inflammatory cell ring two to four cells deep; 4, a ring of inflammatory cells >4 cells deep (Myou et al., 2003).

### Enzyme linked immunosorbent assay (ELISA)

The blood samples collected from the orbital venous plexus and bronchoalveolar lavage fluid (BALF) were placed for 2 h and centrifuged at 4°C for 10 min. The supernatant was obtained and stored at −80°C for later use. OVA-specific IgE, IL-4, IL-5, IL-13, and Interferon gamma (IFN-γ) levels in serum of mice and IL-4, IL-5, IL-13, and IFN-γ contents in BALF were determined by ELISA (Wuhan Eilerite Biotechnology Co., Ltd.). The specific procedures followed the kit’s protocol.

### Western blotting

Using Radioimmunoprecipitation assay buffer (RIPA) lysate, mouse tissues were lysed for 30 min on ice, and protein supernatants were collected by centrifuging at 4 °C and 12,000 rpm for 15 min. The concentration of protein was determined using the BCA assay. The proteins were denatured by adding a loading buffer and heating the mixture for 10 min. The total amount of protein was limited to about 40 μg. After 30 min of electrophoresis at 80 V, the voltage was increased to 120 V for an additional 90 min, and then electroconversion was performed. BSA blocked the PVDF membrane for 2 hours at room temperature. The required antibodies (ERK, proteintech, Cat No. 11257-1-AP, 1:1000; p-ERK, proteintech, Cat No. 28733-1-AP, 1:2000; JNK, proteintech, Cat No. 24164-1-AP, 1:1000, p-JNK, proteintech, Cat No. 80024-1-RR, 1:1000; P38, proteintech, Cat No. 14064-1-AP, 1:1000; p-P38, proteintech, Cat No. 28796-1-AP, 1:1000 and GAPDH, Elabscience, Cat No. E-AB-20059, 1:5000) were diluted in the primary antibody’s dilution solution and incubated overnight at 4°C.After three TBST treatments, the secondary antibody was added and incubated for 2 h at room temperature. The ECL luminescence developer was configured according to specifications, exposed using a chemiluminescence instrument, and the results were analyzed.

### Statistical analysis

Utilizing GraphPad Prism 9 software, the experimental data were analyzed. Multiple groups were compared using a one-way ANOVA with measurement data expressed as mean ± standard deviation (x ± s). Shapiro-Wilk test followed by Tukey’s multiple comparison test was used. The criterion for statistical significance was *p* < 0.05.

## Results

### Prediction of targets and construction of protein interaction (PPI) networks

Initially, we investigated the potential aloin targets using three databases: Swiss Target Prediction, Pharm Mapper, and Similarity Ensemble Approach. Twenty-five genes from the Swiss Target Prediction database, 93 genes from the Pharm Mapper database, and 24 genes from the Similarity Ensemble Approach database were combined to yield 131 drug targets (Figure 1B). The search terms “allergic rhinitis” were entered into Gene Cards, OMIM, TTD, Pharm GKB, and Drug Bank. There were 1977 genes in Gene Cards, 1 in OMIM, 27 in TTD, 9 in Pharm GKB, and 104 in Drug Bank. After combined deduplication, 2047 allergic rhinitis targets were obtained (Figure 1C). Drug and disease targets were combined; eventually, 42 potential aloin-related targets for the treatment of RA were chosen as the primary research foci (Figure 1D). To further investigate the action mechanism of aloin in treating allergic rhinitis, we imported 42 intersection targets into the String database to construct a PPI network graph that contained 41 nodes and 138 edges (Figure 1E). The obtained PPI network map was analyzed by CytoNCA plug-in, with the following selection criteria based on the respective medians: BC > 6, CC > 0.247, DC > 5, EC > 0.078, NC > 3, LAC >2. Gene targets that attained all median criteria were retained and highlighted in Figure 1F, and a new PPI network containing 13 nodes and 72 edges was extracted. Thus, in Figure 1G’s constructed network, only the targets meeting the selection criteria of BC > 1.202, CC > 0.8, DC > 9, EC > 0.290, NC > 8.304, and LAC >7.111 were retained, and a new PPI network consisting of five nodes and ten edges, which was colored yellow, was generated. Figure 1H depicts the final core targets of allergic rhinitis as EGFR, SRC, ESR1, ALB, and MAPK8.

**FIGURE 2:**
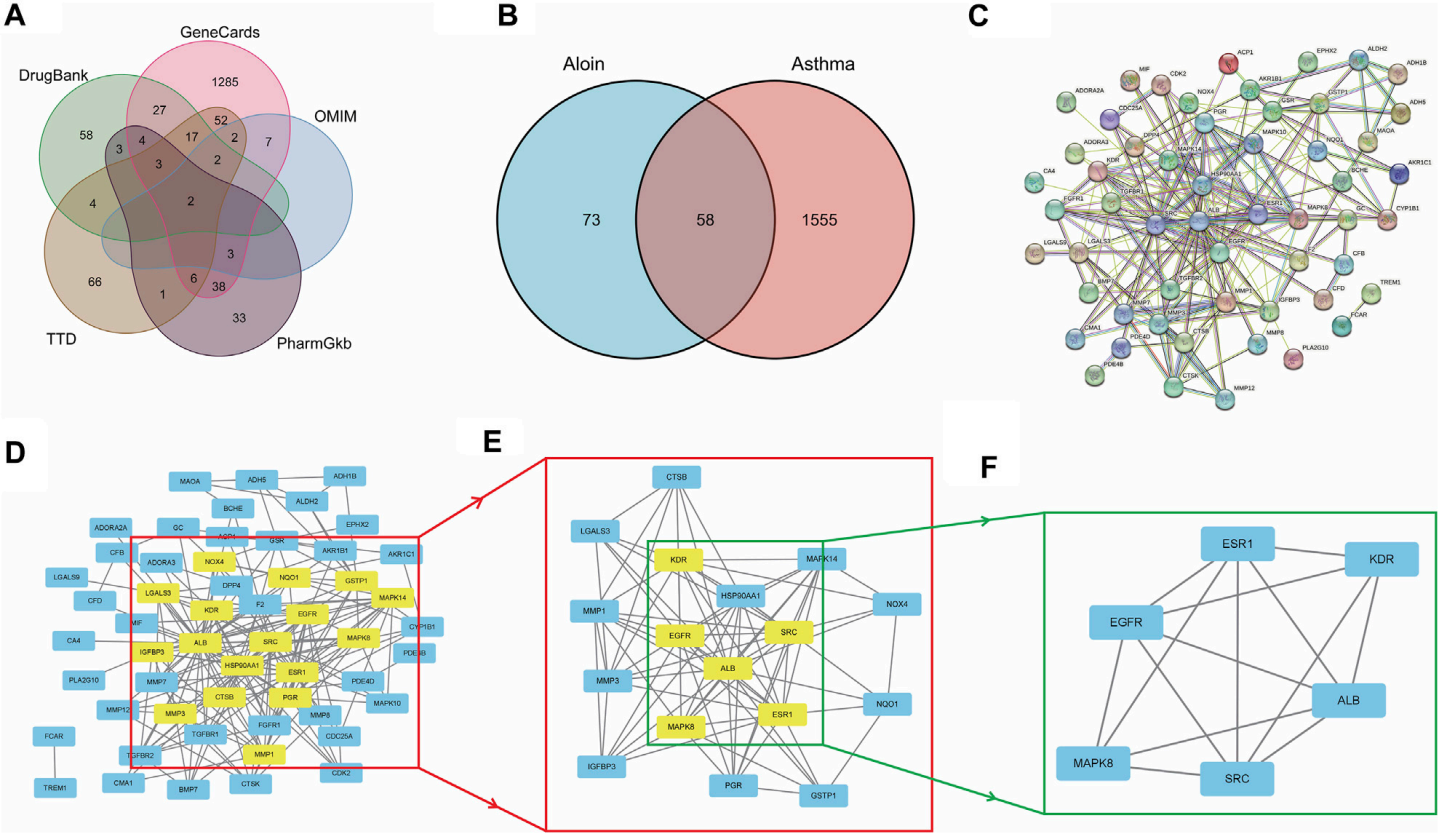
The network pharmacology analysis of Aloin in the treatment of Asthma. **(A)** Potential predictive targets for asthma. **(B)** Aloin and asthma common targets. **(C)** PPI network of common targets. **(D–F)** Topological analysis diagram of the common targets.

Similarly, we sequentially searched five public databases using “asthma” as the search term, including 1448 genes in Gene Cards, 16 in OMIM, 155 in TTD, 93 in Pharm GKB, and 120 in Drug Bank. After combined deduplication, 1613 asthma targets were identified (Figure 2A), and 58 potential targets related to aloin treatment of asthma were obtained after being combined with drug targets, which served as the primary research targets (Figure 2B). Simultaneously, a PPI network was built using 58 intersection targets of aloin in treating asthma, yielding a total of 55 nodes and 205 edges (Figure 2C). The following selection criteria were then applied based on the respective medians: BC > 8.833, CC > 0.244, DC > 5, EC > 0.085, NC > 3.333, LAC >2.333. Gene targets that satisfied all median criteria were retained and highlighted in yellow. A new PPI network with 17 nodes and 78 edges was then extracted (Figure 2D). Consequently, in the network constructed in Figure 2E, only the targets satisfying the selection criteria BC > 3.252, CC > 0.696, DC > 9, EC > 0.234, NC > 7.592, and LAC >6.182 were retained, and a new PPI network with six nodes and 14 edges, highlighted in yellow, was obtained. Figure 2F depicts the core targets determined by topological analysis to be EGFR, SRC, ESR1, ALB, MAPK8, and KDR. Intriguingly, asthma only increased KDR protein compared to AR core targets, and these results strongly suggest that aloin may share similar molecular mechanisms for treating allergic rhinitis and asthma.

### GO and KEGG pathway enrichment analysis

**FIGURE 3:**
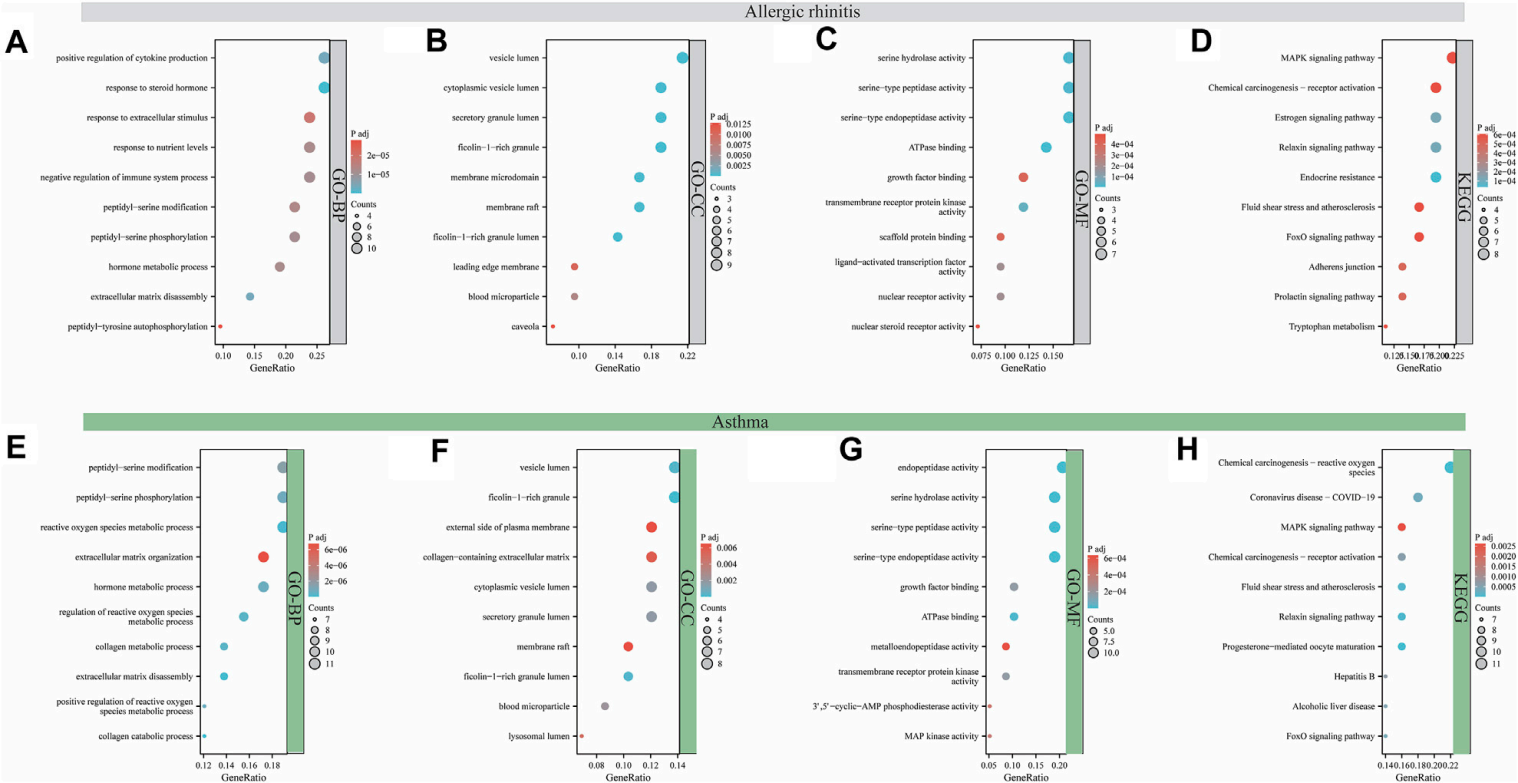
Analysis of GO and KEGG enrichment for target intersection. GO **(A–C)** and KEGG **(D)** enrichment analysis of aloin and AR common targets. GO **(E–G)** and KEGG **(H)** enrichment analysis of aloin and asthma common targets.

The function and signaling pathways of aloin in the treatment of allergic rhinitis and asthma were investigated further. Forty-two intersection aloin targets for treating allergic rhinitis were subjected to GO and KEGG enrichment analyses. The GO results for allergic rhinitis showed a total of 1181 enrichment results. Of these, 1049 were related to BP, 107 to MF, and 25 to CC. The primary biological processes are positive regulation of cytokine production, response to steroid hormone, response to extracellular stimulus, and response to nutrient levels (Figure 3A). The cellular components were predominantly associated with vesicle lumen, cytoplasmic vesicle lumen, secretory granule lumen, and Ficolin-1-rich granule compartments (Figure 3B). Figure 3C illustrates that most molecular functions are associated with serine hydrolase activity, serine-type peptidase activity, serine-type endopeptidase activity, and ATPase binding. Through KEGG pathway enrichment analysis, 57 pathways related to aloin antiallergic rhinitis were identified, and the top 10 pathways with significant enrichment potential were displayed as bubble diagrams in Figure 3D. Mitogen activated protein kinase (MAPK) signaling, chemical carcinogenesis receptor activation, estrogen signaling pathways, and relaxin signaling pathway were the metabolic pathways with the highest enrichment.

Similarly, GO and KEGG enrichment analyses were conducted on the 58 intersection targets of aloin in asthma treatment. Seven hundred fifty-three were associated with BP, 94 were associated with MF, and 19 were associated with CC out of a total of 866 enrichment results for asthma. Figure 3E depicts the biological processes involved in peptidyl-serine modification, peptidyl-serine phosphorylation, reactive oxygen species metabolic process, and extracellular matrix organization. Vesicle lumen, Ficolin-1-rich granule, external side of plasma membrane, and collagen-containing extracellular matrix dominated the cell composition (Figure 3F). The primary molecular functions are endopeptidase activity, serine hydrolase activity, serine-type peptidase activity, and serine-type endopeptidase activity (Figure 3G). KEGG results revealed 82 pathways related to the anti-asthma effects of aloin. The highly enriched metabolic pathways consisted primarily of chemical carcinogenesis reactive oxygen species, coronavirus disease-COVID-19, the MAPK signaling pathway, and chemical carcinogenesis-receptor activation (Figure 3H). These results suggest aloin may be essential in treating allergic rhinitis and asthma via multiple targets and similar signaling pathways.

### Molecular docking validation

**FIGURE 4:**
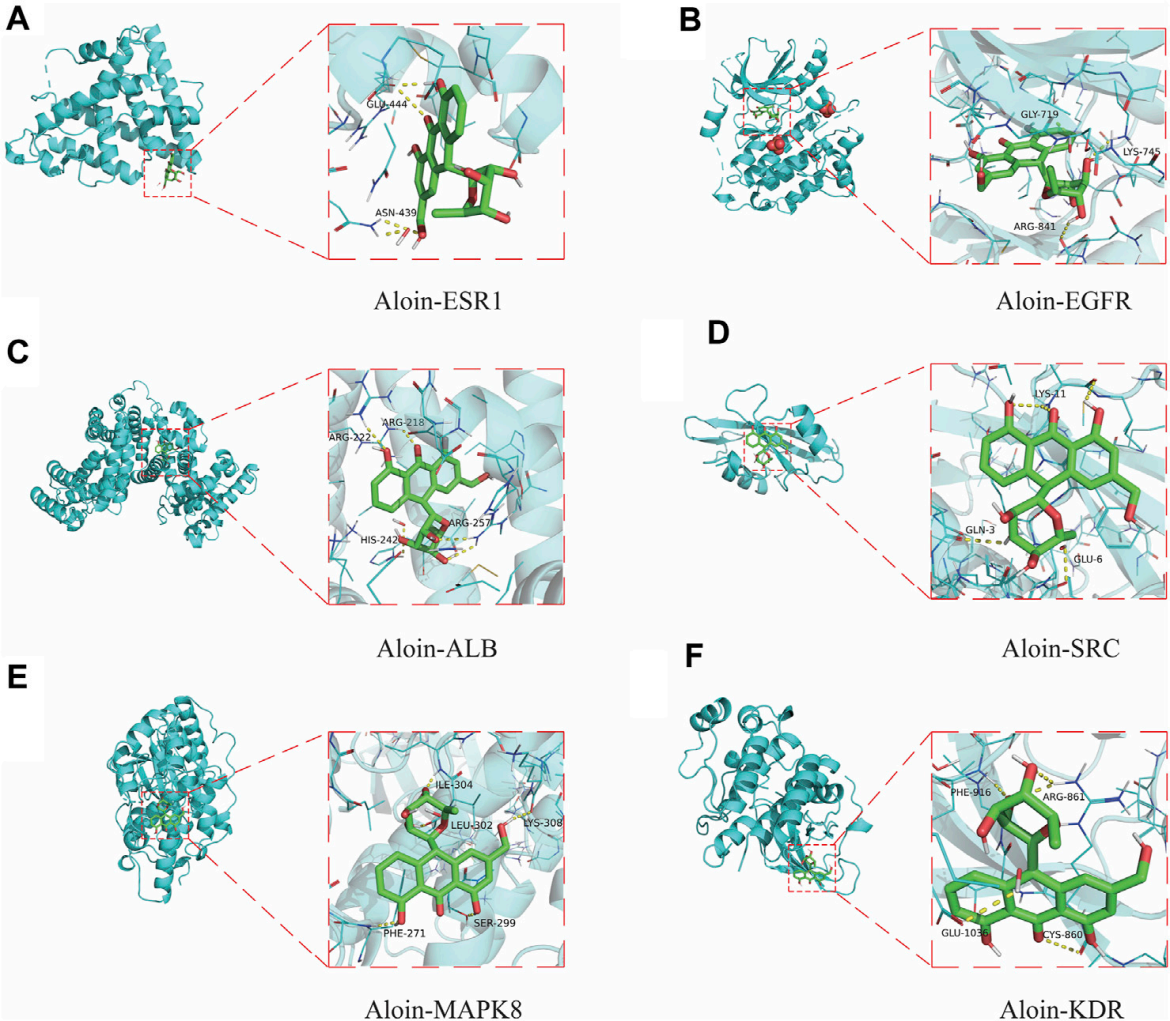
Molecular docking patterns of aloin and core targets. Aloin docked with **(A)** ESR1, **(B)** EGFR, **(C)**ALB, **(D)**SRC, **(E)** MAPK8, **(F)** KDR.

To further validate the predictive ability of bioinformatics, molecular docking was used to examine aloin’s potential for treating AR and asthma. The AR core targets (EGFR, SRC, ESR1, ALB, and MAPK8) were selected for molecular docking with aloin following Cyto NCA analysis. The results indicated aloin could interact with ESR1’s GLU-444 and ASN-439 via two hydrogen bonds (Figure 4A). It could form a single hydrogen bond with GLY-719, LYS-745, and ARG-841 of EGFR (Figure 4B). It can form two hydrogen bonds with ALB’s ARG-257 and one hydrogen bond with ARG-218, ARG-222, and HIS-242 (Figure 4C). Figure 4D depicts the formation of three, one, and one hydrogen bonds between LYS-11, GLU-3, and GLU-6 in SRC. One hydrogen bond in MAPK8 allows aloin to interact with PHE-271, SER-299, LEU-302, ILE-304, and LYS-308 (Figure 4E). The core asthma targets (EGFR, SRC, ESR1, ALB, MAPK8, and KDR) were simultaneously selected for molecular docking with aloin. Since asthma has only more KDR proteins than AR, the rest of the molecular docking is the same as previously described, and aloin can form two hydrogen bonds with ARG-861, PHE-916, GLU-1036, and CYS-860 in KDR (Figure 4F). Thus, there may be an interaction between aloin, AR, and asthma via hydrogen bonding.

Frequently, binding energy is used to characterize the degree of affinity between a receptor and ligand. The lower the binding energy, the greater the affinity and the more stable the conformation. Generally, binding energies less than −5.0 kcal/mol or −7.0 kcal/mol indicate good or strong binding activity between ligand and receptor, respectively. The results indicated that the binding free energies of EGFR, ALB, KDR, MAPK8, ESR1 and SRC to the aloin molecules were −9.8, −8.6, −7.9, −7.4, −7.0, and −6.4, respectively. The above molecules’ docking binding free energies were all ≤ −5; hence, aloin appears to have a high affinity for the primary protein targets of AR and asthma.

### Molecular dynamics simulation

Using Gromacs-2022.04, we simulated the molecular dynamics (MD) of a 100 ns protein-ligand complex for EGFR, ALB, MAPK8, ESR1, and SRC, respectively, which are typical core targets for allergic rhinitis and asthma. In addition, analyses were conducted on the RMSD, RMSF, Rg, Hbond, and SASA. In order to assess the stability of the ligand-protein complex, the ligand was compared to the corresponding reference ligand.

**FIGURE 5:**
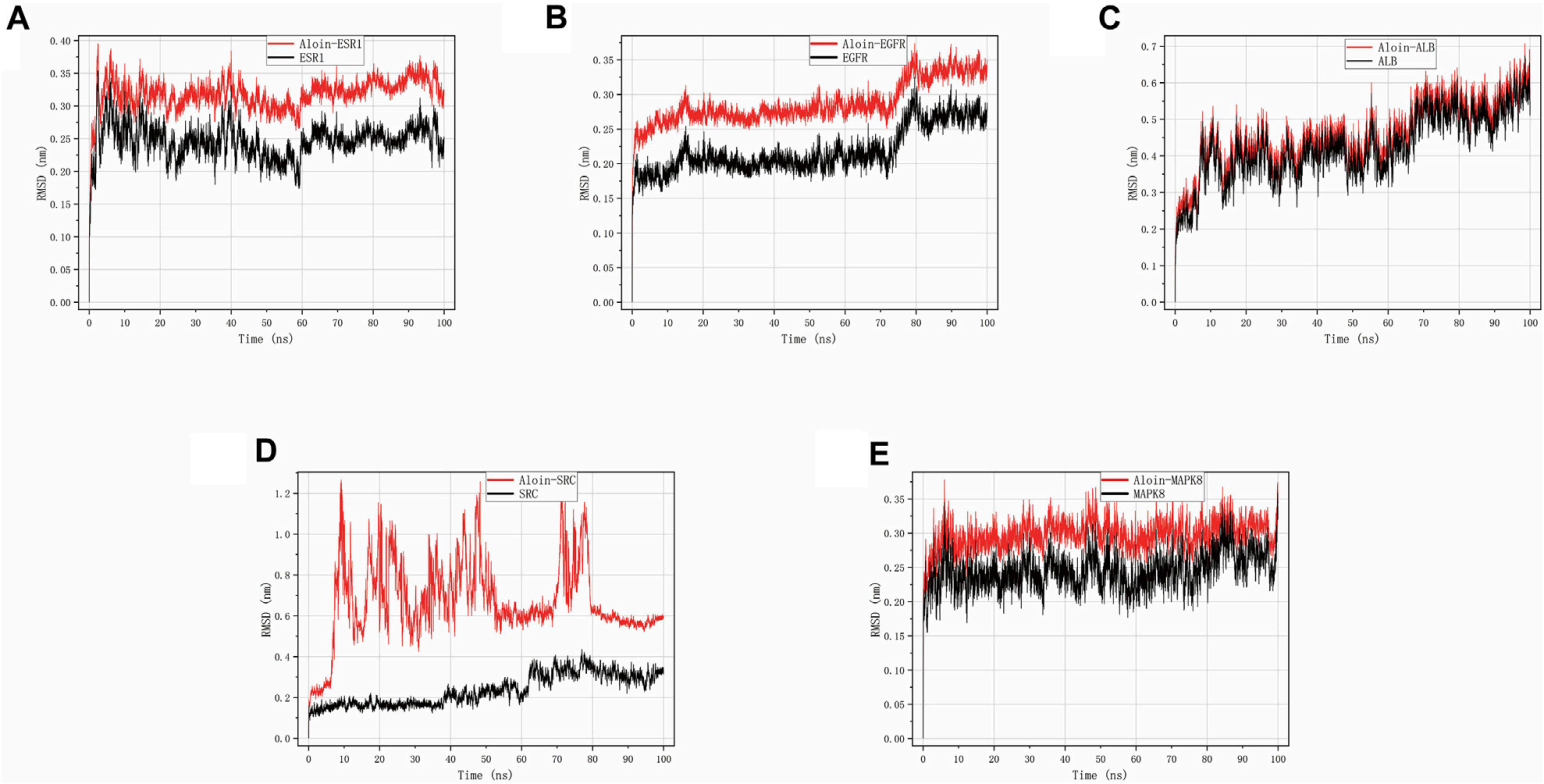
MD simulates RMSD trace values of protein-ligand complexes. Aloin and **(A)** ESR1, **(B)** EGFR, **(C)**ALB, **(D)**SRC, **(E)** MAPK8 RMSD trace.

RMSD can disclose the positional difference between the protein’s simulation-generated conformation and its initial conformation. Aloin-ESR1 (Figure 5A) and aloin-MAPK8 (Figure 5E) were stable for approximately 10 ns, according to the results. After some fluctuation at 70 ns, aloin-EGFR reached a relatively stable state at approximately 17 ns and then stabilized at 80 ns (Figure 5B). Aloin-ALB stabilized at approximately 10 ns, fluctuated at 60 ns, and stabilized at 70 ns (Figure 5C). Aloin-SRC fluctuated initially and stabilized after 80 ns, but it fluctuated more than the reference ligand (Figure 5D). These results indicate that the conformation of most proteins does not significantly change after aloin binds to them relative to the reference ligand and that the binding between the two proteins is relatively stable.

**FIGURE 6:**
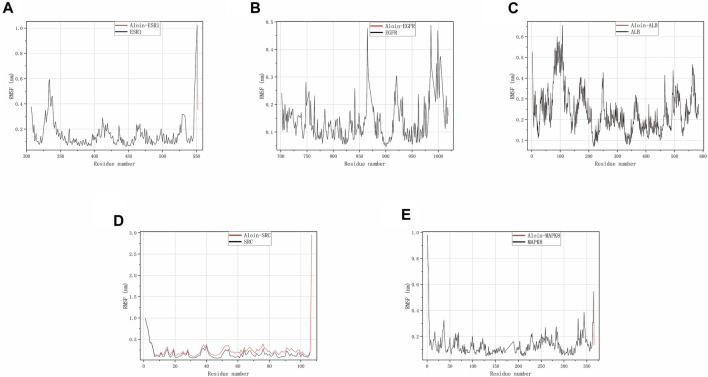
MD simulates RMSF trace values of protein-ligand complexes. Aloin and **(A)** ESR1, **(B)** EGFR, **(C)** ALB, **(D)** SRC, **(E)** MAPK8 RMSF trace.

Throughout the simulation, RMSF characterizes the flexibility and motion intensity of protein amino acids. As shown in the figure, the drug binds to the protein to stabilize it and exert enzyme activity, which generally reduces the protein’s flexibility. Compared to the corresponding reference ligand, all complexes exhibit a similar tendency, i.e., they cause some fluctuation in the same protein region (Figures 6A–E). However, all compounds generally have high rigidity and structural stability.

**FIGURE 7:**
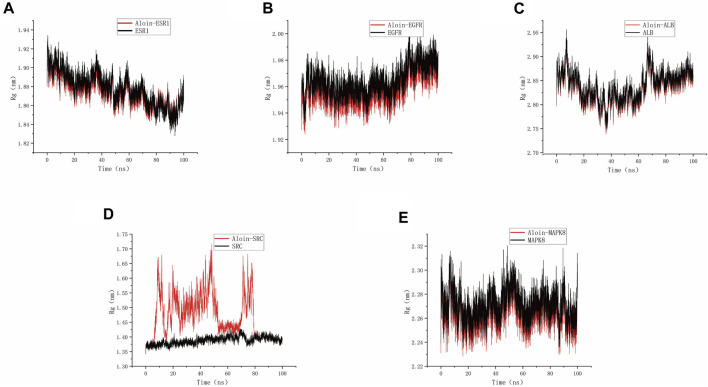
MD simulates Rg trace values of protein-ligand complexes. Aloin and **(A)** ESR1, **(B)** EGFR, **(C)**ALB, **(D)**SRC, **(E)** MAPK8 Rg trace.

As shown in the figure, Rg can be used to represent the compactness of the protein structure. Throughout the simulation, the structural dynamics of aloin-EGFR, aloin-ESR1, aloin-MAPK8, and aloin-ALB complexes remained relatively stable (Figures 7A–C,E). Only aloin-SRC fluctuated, and the Rg value was biased (Figure 7D), indicating that aloin was strongly bound to most proteins.

**FIGURE 8:**
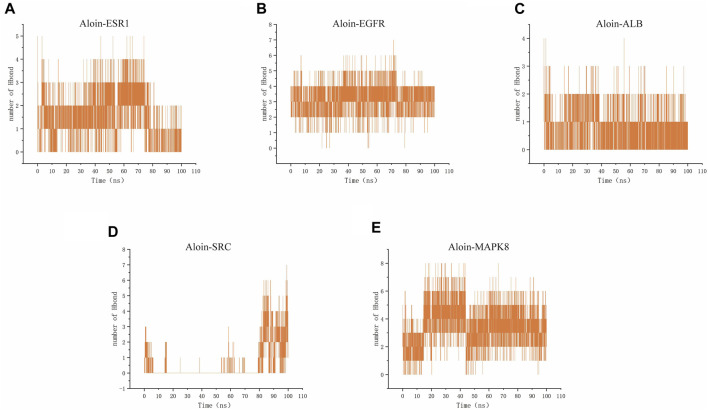
MD simulates the number of hydrogen bonds of protein-ligand complexes. Number of Aloin and **(A)** ESR1, **(B)** EGFR, **(C)**ALB, **(D)**SRC, **(E)** MAPK8 Hbond.

Hydrogen bonding is one of the strongest non-covalent binding interactions, making comprehending the binding affinity between ligands and proteins essential. The results indicated that the hydrogen bond numbers for the aloin-ESR1, aloin-EGFR, aloin-MAPK8, aloin-ALB, and aloin-SRC complexes were 0–5, 0–7, 0–8, 0–5, 0–4, and 0–7, respectively (Figures 8A–E). It indicates that the number of hydrogen bonds formed by all protein-ligand complexes remains unchanged throughout the simulation. In addition, persistent amino acid residues at the active site contribute to the overall structural stability of the complex.

**FIGURE 9:**
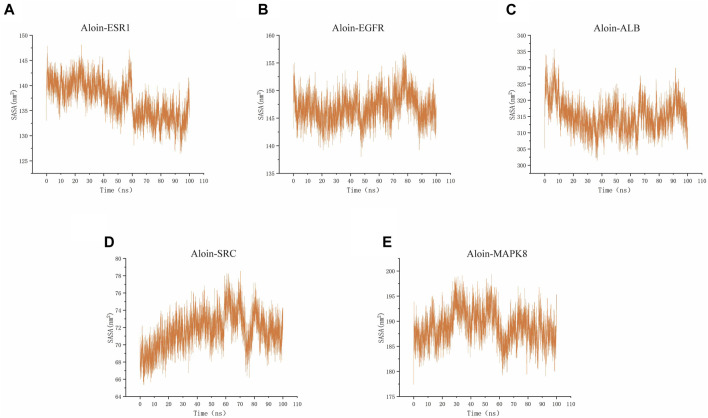
MD simulates SASA of protein-ligand complexes. Aloin and **(A)** ESR1, **(B)** EGFR, **(C)**ALB, **(D)**SRC, **(E)** MAPK8 SASA trace.

SASA is a valuable parameter for studying protein conformational dynamics in solvent environment. The results indicated that the five complexes had similar contact areas with water, while small molecules had little effect on protein-water action (Figures 9A–E). The above MD simulation results depicted that the binding of aloin to most proteins was relatively stable.

### Aloin alleviates the clinical symptoms of CARAS mouse

**FIGURE 10:**
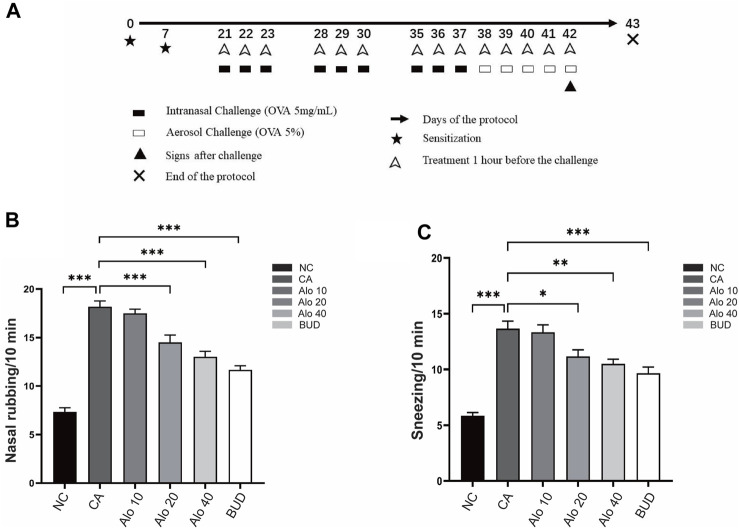
Construction and validation of the CARAS mouse model.**(A)** Flow chart of CARAS mouse model construction. The number of nasal rubbing **(B)** and sneezing **(C)** in each group was counted. *, *p* < 0.05; **, *p* < 0.01; ***, *p* < 0.001.

To examine the anti-inflammatory effects of aloin on AR and asthma, mouse induced by OVA were used as animal models of CARAS (Figure 10A), and various concentrations of aloin were chosen for animal experiments. The number of sneezes and nasal rub was substantially greater in the CARAS group than in the control. However, The number of nasal symptoms decreased significantly in the medium-dose group (20 mg/kg) and the high-dose group (40 mg/kg) and BUD group (Figures 10B,C). These results indicate that Alon significantly alleviated clinical symptoms in CARAS mouse.

### Aloin reduces inflammatory infiltration in nasal mucosa and lung tissue of CARAS mouse

**FIGURE 11:**
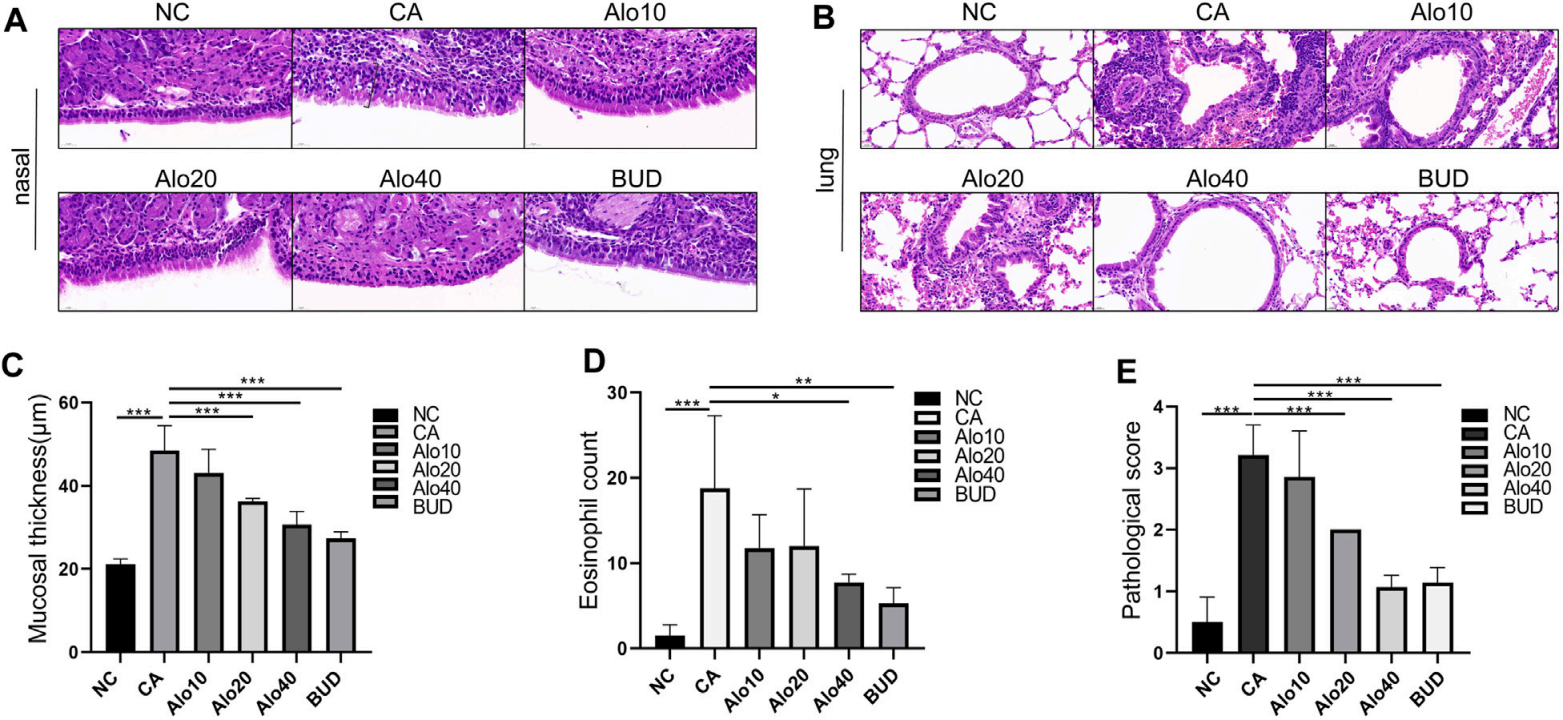
Histological analysis of nasal and lung tissues. **(A)** H&E-stained nasal tissue. (Black square brackets indicate mucosal thickness). **(B)** H&E-stained lung tissue. **(C)** Quantitative count of eosinophils. **(D)** Quantitative statistics of mucosal thickness. **(E)** Lung inflammation score. Scale bar = 20 μm *, *p* < 0.05; **, *p* < 0.01; ***, *p* < 0.001.

**FIGURE 12:**
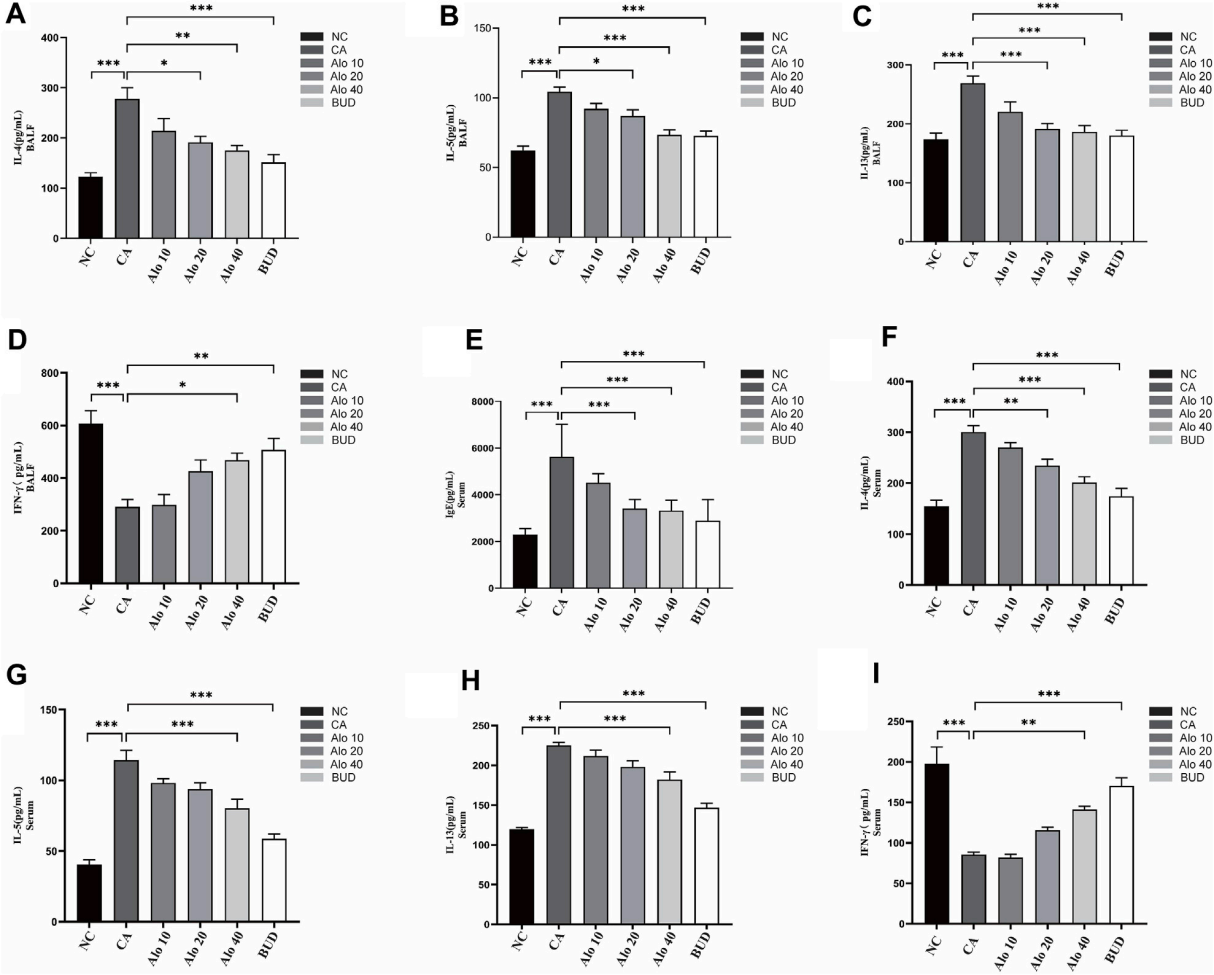
Expression levels of inflammatory factors in mice with CARAS treated with aloin. **(A–D)** ELISA was used to detect the levels of IL-4, IL-5, IL-13 and IFN-γ in BALF, and **(E–I)** detected IgE, IL-4, IL-5, IL-13 and IFN-γ in serum. *, *p* < 0.05; **, *p* < 0.01; ***, *p* < 0.001.

Subsequently, we investigated further the effect of aloin on the inflammatory infiltration of CARAS mouse nasal mucosa and lung tissue. The thickness of nasal mucosa and the degree of bronchial inflammatory infiltration in the CARAS group were significantly higher than those in the other groups. The thickness of nasal mucosa and the degree of inflammatory infiltration in the middle-dose aloin group (20 mg/kg) and the high-dose aloin group (40 mg/kg) and BUD group were less than the CARAS group (Figures 11A–C). Furthermore, the mouse in the CARAS group displayed a high number of eosinophil infiltration in the nasal mucosa and lung tissue. Aloin medium and high dose (20, 40 mg/kg) treatment and BUD group demonstrated less eosinophil infiltration compared to the CARAS group (Figure 11D). In addition, we evaluated the effect of aloin on the concentrations of inflammatory factors in CARAS. The levels of IL-4, IL-5, and IL-13 in serum and BALF and total IgE were significantly increased in CARAS group, while they were significantly decreased in the high-dose aloin group (40 mg/kg) and BUD group. In contrast, the IFN-γ significantly decreased, in CARAS group, while it was significantly increased in the high-dose aloin group (40 mg/kg) and BUD group (Figures 12A–D, 12F–12I). The CARAS group had significantly higher serum OVA-specific IgE levels than the control group, whereas aloin medium and high dose (20, 40 mg/kg) treatment and BUD group decreased OVA-specific IgE immunoglobulin production (Figure 12E). These results demonstrated that aloin significantly decreased inflammatory infiltration in CARAS.

### Aloin can inhibit the inflammatory level of CARAS through the MAPK pathway

**FIGURE 13:**
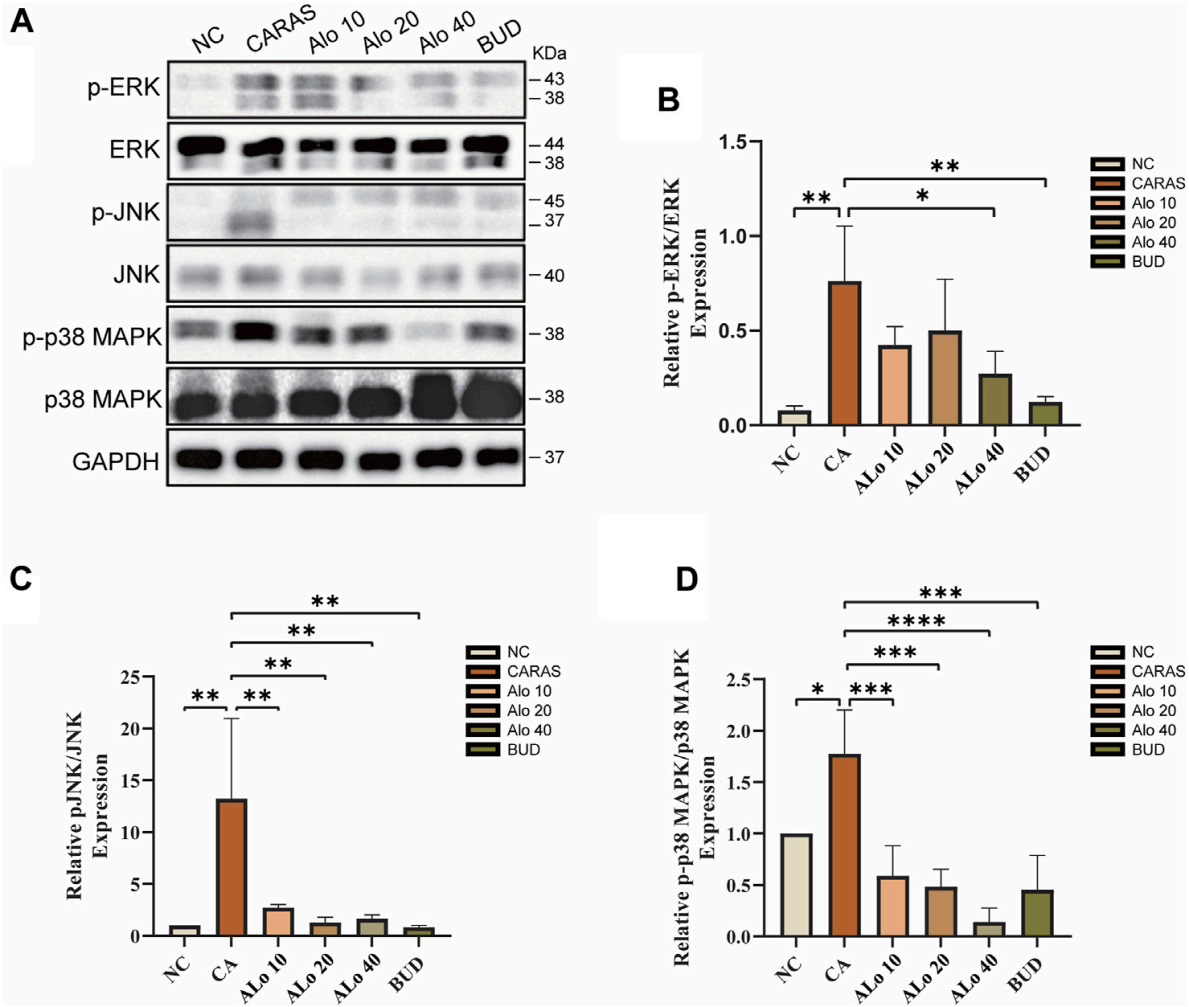
Expression levels of MAPK pathway-related proteins in Aloin treated CARAS mice. **(A)** Representative band diagram of MAPK pathway-related proteins. **(B)** Aloin treatment reduced p-ERK expression levels. **(C)** Aloin treatment reduced the expression level of p-JNK. **(D)** Aloin treatment reduced the expression level of p-P38/MAPK. *p < 0.05, **p < 0.01, ***p < 0.001.

Next, we investigated the activity of aloin resistance CARAS potential mechanism. KEGG enrichment analysis indicated that aloin might significantly treat allergic rhinitis and asthma via the MAPK signaling pathway. Western blot was used to detect the expression of MAPK signaling pathway-related proteins p-ERK, p-JNK, and p-p38 MAPK in mouse tissues (Figure 13A). The levels of phosphorylated MAPK family members (ERK, JNK, and p-p38) were significantly elevated in the CARAS group, whereas aloin medium and high dose (20, 40 mg/kg) treatment and budesonide treatment significantly inhibited ERK activation. pERK expression was not significantly reduced in the low-dose (10 mg/kg) group (Figure 13B). In addition, treatment with different doses of aloin (10, 20, and 40 mg/kg) and budesonide inhibited JNK and p-p38 activation significantly (Figures 13C,D). These results suggest that aloin may suppress the inflammatory response in CARAS by modulating the MAPK signaling pathway.

## Discussion

Connecting the upper and lower airways, CARAS is a chronic inflammatory disease. Studies on the physiopathology of allergic rhinitis and asthma have revealed that they are triggered by the same identical nature and etiology and have profound correlations, with a comparable spectrum of cellular and humoral immune responses to mediate the inflammatory process. Although many drugs, such as glucocorticoids and antihistamines, are used as antiallergic therapy to alleviate the symptoms of these two diseases, long-term treatment can cause potentially serious side effects and diminish the desired effect (Li et al., 2018; Xia et al., 2018). Thus, there is an immediate need to develop a new, safer, and more effective CARAS treatment. Network pharmacology is a strategy for network analysis of biological systems based on high-throughput omics data analysis, virtual computer computation, and network database retrieval. Based on the theories of systems biology, multi-directional pharmacology, genomics, proteomics, and other disciplines, and the technologies of omics, high-throughput screening, network visualization, and network analysis, the multi-level and multi-angle biological network relationship between “drug-gene-target-disease” is revealed and analyzed to anticipate potential drug action mechanism and render an essential resource for determining the pharmacological effectiveness and mechanism of drugs. As the primary bioactive component of aloe vera, aloin possesses a broad spectrum of pharmacological activities, such as anti-tumor, anti-inflammatory, antiviral, antimicrobial, lipid, and glucose regulation. It has the potential to be developed into good healthcare products (Dai et al., 2022).

Through network pharmacology, molecular docking, molecular dynamics simulation, and experimental verification via animal models, we demonstrated for the first time that aloin has an anti-inflammatory effect on CARAS and that its protective effect may be achieved by regulating the MAPK signaling pathway. These findings provide direction for a future experimental investigation into the molecular mechanism of aloin in the ameliorates of CARAS. First, the potential therapeutic targets of aloin in treating allergic rhinitis and asthma were extracted from various public databases, followed by the construction of a protein network interaction analysis. Key targets for allergic rhinitis and asthma were EGFR, SRC, ESR1, ALB, and MAPK8, and allergic rhinitis and asthma syndrome were the “same airway disease.” In conclusion, EGFR, SRC, ESR1, ALB, and MAPK8 are most likely the primary targets of aloin in inhibiting CARAS and play a crucial role in reducing CARAS inflammation. These proteins play crucial roles in inflammatory responses, mucus secretion, and numerous other cellular processes that are believed to be essential for treating CARAS (Dijkstra et al., 2006; Kumar et al., 2015; Jia et al., 2021; Yang et al., 2022). KEGG enrichment analysis revealed that MAPK was a common signaling pathway in treating allergic rhinitis and asthma. The MAPK pathway has three major branches: ERK, JNK, and p38/MAPK (Yi et al., 2020). JNK and p38 have similar functions associated with inflammation, apoptosis, and cell growth. Ras/Raf proteins are the upstream signal for ERK, which regulates the growth and differentiation of tube cells. By phosphorylating nuclear transcription factors, cytoskeletal proteins, and enzymes, MAPK is involved in the regulation of cell proliferation, differentiation, transformation, and apoptosis and is closely associated with the occurrence of inflammation, cancer, and other diseases. According to several studies, MHTP, as a synthetic alkaloid, alleviates CARAS by down-regulating the p38/MAPK signaling pathway in mice (Paiva Ferreira et al., 2021). These results may indicate a regulatory mechanism by which aloin inhibits the progression and development of CARAS. Molecular docking and molecular dynamics simulations were then used to investigate further the stability and interaction of aloin and its target proteins. Aloin demonstrated the ability to bind to core targets, and the stable molecular docking model demonstrated a strong and efficient binding between aloin and core targets. During the 100 ns MD simulation, the conformation of the complex did not change significantly, indicating that aloin binding remained relatively stable. These results strongly confirmed that aloin has a potential curative effect on CARAS.

In the CARAS mouse model, we observed significant symptoms such as nose scratching and sneezing, however aloin could significantly relieve nasal symptoms. Meanwhile, histopathological analysis showed that aloin treated CARAS mouse had thinner nasal mucosa and reduced peribronchial inflammatory infiltration. These findings proved the efficacy of aloin in alleviating OVA-induced inflammatory response. Moreover, the imbalance between Th 1 and Th 2 cell-mediated immunity plays a crucial role in the pathophysiology of CARAS. The infiltration of eosinophils and a polarized type 2 immune response producing inflammatory cytokines influence inflammatory progression. In the present study, aloin treated group reduced IL-4, IL-5 and IL-13 levels in BALF and increased the levels of IFN-γ, which affected IgE synthesis and the growth and differentiation of eosinophils. Interestingly, the same trend was observed for cytokines in mouse serum, further confirming the effect of aloin. Additionally, protein interaction network topology analysis, KEGG enrichment analysis, molecular docking, and molecular dynamics simulation indicated aloin might act on CARAS via the MAPK pathway. In this study, CARAS mouse had elevated phosphorylated p38, ERK, and JNK. However, after the administration of aloin, the MAPK pathway-related protein levels were reversed. The current study found that aloin may diminish airway inflammation by reducing the expression of phosphorylated proteins in the MAPK signaling pathway. Nevertheless, there are limitations to this research. The mechanism of aloin against CARAS remains unconfirmed, and additional *in vitro* experiments are required to investigate the molecular mechanism of aloin in treating CARAS.

## Conclusion

According to the findings of this study, aloin may ameliorate OVA-induced CARAS by modulating the MAPK signaling pathway. Aloin may be a promising treatment option for CARAS. This research provides a firm basis for comprehending the mechanism of aloin and its clinical application in treating CARAS.

## Figures and Tables

**Table:** 

Al(OH)3	Aluminium hydroxide
AR	Allergic rhinitis
BALF	bronchoalveolar lavage fluid
BC	betweenness
BP	Biological process
BUD	Budesonide
CARAS	The combined allergic rhinitis and asthma syndrome
CC	Cellular Component
CC	closeness
DC	degree
EC	Eigenvector
ELISA	Enzyme linked immunosorbent assay
ELISA	Enzyme-linked Immunosorbent Assay
GAFF	The generalized Amber force field
GO	Gene ontology
H&E	Hematoxylin-Eosin staining
Hbond	Hydrogen bond
IFN-γ	Interferon gamma
IgE	Immunoglobulin E
IL-13	Interleukin-13
IL-4	Interleukin-4
IL-5	Interleukin-5
KEGG	Kyoto encylopaedia of genes and genomes
LAC	Local Average Connections-based
MAPK	Mitogen activated protein kinase
MD	molecular dynamics
MF	Molecular function
MF	Molecular function
NC	Network
NVT	canonical ensemble
OMIM	Online mendelian inheritance in man
OVA	Ovalbumin
PDB	Protein Data Bank
PPI	protein interaction
Rg	The radius of gyration
RIPA	Radioimmunoprecipitation assay buffer
RMSD	The root mean square deviation
RMSF	Root mean square fluctuation
SASA	solvent accessible surface
SPF	Specific pathogen Free
TTD	Therapeutic target database
Uniprot	Universal Protein Resource

## Data Availability

The original contributions presented in the study are included in the article/Supplementary Materials, further inquiries can be directed to the corresponding author.
